# The Integrity of the Blood–Brain Barrier as a Critical Factor for Regulating Glutamate Levels in Traumatic Brain Injury

**DOI:** 10.3390/ijms24065897

**Published:** 2023-03-20

**Authors:** Matthew Boyko, Benjamin F. Gruenbaum, Dmitry Frank, Dmitry Natanel, Shahar Negev, Abed N. Azab, Guy Barsky, Boris Knyazer, Ora Kofman, Alexander Zlotnik

**Affiliations:** 1 Department of Anesthesiology and Critical Care, Soroka University Medical Center, Ben-Gurion University of the Negev, Beer-Sheva 84101, Israel; 2 Department of Anesthesiology and Perioperative Medicine, Mayo Clinic, Jacksonville, FL 32224, USA; 3 Department of Nursing, Recanati School for Community Health Professions, Faculty of Health Sciences, Ben-Gurion University of the Negev, Beer-Sheva 84101, Israel; 4 Department of Surgery B, Soroka University Medical Center and the Faculty of Health Sciences, Ben-Gurion University of the Negev, Beer-Sheva 84101, Israel; 5 Department of Ophthalmology, Soroka University Medical Center and the Faculty of Health Sciences, Ben-Gurion University of the Negev, Beer-Sheva 84101, Israel; 6 Department of Psychology, Zlotowski Center for Neuroscience, Ben-Gurion University of the Negev, Beer-Sheva 84101, Israel

**Keywords:** blood–brain barrier (BBB), glutamate, neurotoxicity, rats, traumatic brain injury (TBI)

## Abstract

A healthy blood–brain barrier (BBB) shields the brain from high concentrations of blood glutamate, which can cause neurotoxicity and neurodegeneration. It is believed that traumatic brain injury (TBI) causes long-term BBB disruption, subsequently increasing brain glutamate in the blood, in addition to increased glutamate resulting from the neuronal injury. Here, we investigate the relationship between blood and brain glutamate levels in the context of BBB permeability. Rats exposed to BBB disruption through an osmotic model or TBI and treated with intravenous glutamate or saline were compared to control rats with an intact BBB treated with intravenous glutamate or saline. After BBB disruption and glutamate administration, the concentrations of glutamate in the cerebrospinal fluid and blood and brain tissue were analyzed. The results showed a strong correlation between the brain and blood glutamate concentrations in the groups with BBB disruption. We conclude that a healthy BBB protects the brain from high levels of blood glutamate, and the permeability of the BBB is a vital component in regulating levels of glutamate in the brain. These findings bring a new approach to treating the consequences of TBI and other diseases where long-term disruption of the BBB is the central mechanism of their development.

## 1. Introduction

Traumatic brain injury (TBI) is associated with neurologic and psychiatric effects, including depression, anxiety, and aggression [1,2,3,4,5]. Although the psychiatric symptoms were presumed to be related to the emotional toll of TBI-related physical disability, neuropsychiatric symptoms, including memory and cognitive impairment, anxiety, depression, social withdrawal or aggression [6,7,8,9], have been shown to persist decades [10,11] after the initial brain insult. These neuropsychiatric effects affect recovery [12] and are not dependent on the severity of the initial injury or pain level [13]. The mechanisms underlying the development of neuropsychiatric disorders after TBI remain unclear. In this study, we investigate the relationship between brain and blood glutamate levels in the context of blood–brain barrier (BBB) permeability. We hypothesize that prolonged dysregulation of glutamate, caused by alterations in the permeability of the BBB following TBI [14,15], can trigger neurodegeneration and subsequently contribute to the development of neuropsychiatric disorders.

Glutamate is a free amino acid in the brain [16], with a concentration in the plasma and whole blood of 50–100 μM/L and 150–300 μM/L, respectively [17], and in the whole brain of 10,000–12,000 μM/kg [18], but only 1–10 μM/L in extracellular fluids (ECF) [17]. The gradient between brain cells, blood, and ECF is supported by the facilitative and active transport systems of the BBB [15]. An unimpaired BBB successfully stops glutamate from traveling through the intraparenchymal and blood compartments [19]. It seems clear that the factors of BBB destruction have a significant impact on increasing brain glutamate and have an association with TBI [15]. We have identified a number of mechanisms associated with TBI that increase glutamate in the brain [15], including neuronal death, inflammation, impaired glutamatergic recycling and signaling, prolonged stress, astrocytic release of adenosine triphosphate (ATP), and other sources of high intraparenchymal glutamate [15].

Disruption of the BBB has a crucial role to play in producing higher levels of brain glutamate in the CSF and ECF. The peak of the destruction of the BBB after brain insult occurs 5–6 h after the injury [20]. Studies indicate that the integrity of the BBB in rats may not be restored until 1–3 months [21] after the initial brain injury, with some reporting as long as 10 months before restoration [22]. In humans, restoration can take years [20]. It has been established that a damaged BBB cannot assist in the successful clearance of cerebral glutamate from ECF into the bloodstream, with a possible effect on the ability of excitatory amino acid transporters (EAAT) on endothelial cells to modulate the intraparenchymal-blood glutamate concentration gradient [19].

We have recently hypothesized that the integrity of the BBB is partially responsible for limiting pathologically increased levels of ECF and CSF glutamate in the post-TBI phase of neuronal death and establishes the maximal level of glutamate in the brain after TBI [15]. In addition, BBB integrity also exerts control over the minimal level of brain glutamate after TBI [15]. Low glutamate levels are normally mediated by facilitative and active transport systems of an intact BBB in an unimpaired brain, moving glutamate from the brain into the blood [23]. With disruption in BBB integrity, however, this system is also disrupted, and glutamate cannot be transported from the CSF and ECF into the blood, thereby altering the concentration gradient in the blood. 

Despite the wide spectrum of sensitivity of nervous tissue to high concentrations of glutamate, data suggest that even a slight increase of 10% of extracellular brain glutamate (glutamate neurotoxicity) over a long period of time can activate neurodegenerative cascades [16,17]. Therefore, the integrity of the BBB is a major aspect in determining the range of glutamate concentration in intact and compromised brains. 

Glutamate neurotoxicity involves excessive glutamate that induces neuronal degeneration and dysfunction, causing neurotoxicity [16,24,25]. Increased glutamate levels have also been associated with seizure induction, especially in the limbic system [26]. Recent research on mood disorders has used this association to target glutamatergic systems as pathways for new therapeutic modalities for anti-depressants and related therapies [27,28,29]. 

Following acute brain injury, such as ischemic strokes, cerebral ischemia, TBI, hypoglycemia, and epilepsy, increased extracellular glutamate can produce excitotoxicity due to the overaction of ionotropic glutamate receptors [16,30,31,32]. It is also likely that glutamate neurotoxicity can have a chronic effect, as it may play a similar role in neurodegenerative diseases, including amyotrophic lateral sclerosis, Alzheimer’s disease and Huntington’s disease [31]. Chronic excitotoxicity could occur in diseases where nerve cell death happens over an extended length of time, with the neurons exposed to glutamate at high levels gradually suffering from cell death [31]. We propose that treatments for these diseases may be effective because they restore glutamatergic homeostasis through initiating glutamate uptake and extruding extracellular glutamate. The progression outlined here of glutamate neurotoxicity and its effect on neurodegeneration suggests that effective new treatments for acute and chronic neurological diseases could target the glutamatergic system. 

The main purpose of this manuscript is to investigate the role of BBB disruption as a factor that contributes to excess glutamate through a rat model of TBI and model of BBB disruption. We, thus, outline the proposed pathways of the process and supply evidence to demonstrate this association. We propose that this relationship provides a useful tool to understanding the mechanisms for the possible treatment of TBI and its sequelae.

## 2. Results

### 2.1. Neurological Performance

There was no evidence of neurological deficit in the control group. The NSS at 5 h was significantly greater in TBI rats compared to the control group (10(7–13) vs. 0(0–0), U = 0, *p* < 0.01, r = 0.749). The data were measured as a count and expressed as the median and 25–75 percentile range (Mann–Whitney *U* test). No significant difference was observed between the males and females.

### 2.2. Main and Interaction Effects on Outcomes

We employed a 3-way ANOVA to determine the main effects of the model of BBB disruption (TBI at 6 h, TBI at 24 h, HOBBB, or sham), treatment (glutamate 1.69 g/kg, glutamate 0.845 g/kg, glutamate 0.423 g/kg or placebo), and sex (male or female) on outcomes such as blood glutamate concentration, CSF glutamate concentration, and Evans blue extravasation index.

#### 2.2.1. Evans Blue Extravasation Index

A 3-way ANOVA showed a significant effect of BBB disruption (F_3,199_ = 270, *p* < 0.01, _p_η^2^ = 0.818). A Bonferroni post-hoc analysis showed a significantly higher Evans blue extravasation index (for rats that underwent the BBB procedure with TBI at 6 h (82 × 10^−7^ g *±* 30 × 10^−7^ g, *p* < 0.01), TBI at 24 h (25 × 10^−7^ g *±* 10 × 10^−7^ g, *p* < 0.01), and HOBBB (9.5 × 10^−7^ g *±* 3.2 × 10^−7^ g, *p* < 0.01) compared to the control rats (−0.055 × 10^−7^ g ± 0.187 × 10^−7^ g). Our analysis found no significant effects of sex and treatment, and no influence on BBB disruption (F < 1 in all cases). Since no sex or treatment differences were found in the Evans blue extravasation index, an initial t-test for the study groups was performed and confirmed that sex and treatment did not affect the Evans blue extravasation index. The data are measurements in grams of extravasated Evans blue dye per gram of brain tissue and expressed as mean ± SD (see Table 1).

#### 2.2.2. Blood Glutamate Level

A 3-way ANOVA showed a significant effect on blood glutamate levels (F_3,199_ = 779, *p* < 0.01, _p_η^2^ = 0.929). Post-hoc analysis with a Bonferroni test denoted a significantly higher level of blood glutamate for rats receiving glutamate 0.423 g/kg (927 µM/L ± 251 µM/L, *p* < 0.01), glutamate 0.845 g/kg (1361 µM/L ± 189 µM/L, *p* < 0.01) and glutamate 1.69 g/kg (1726 µM/L ± 275 µM/L, *p* < 0.01) compared to the placebo (224 µM/L ± 26 µM/L). No effects of sex and severity of BBB disruption or interaction between sex and severity of BBB disruption on blood glutamate levels were found (F < 1 in all cases). Since no difference in sex or severity of BBB disruption was found in blood glutamate levels, an initial t-test for the study groups confirmed the result that sex and severity of BBB disruption did not demonstrate a significant difference in blood glutamate levels. The data are measurements in µM/L and expressed as a mean ± SD (see Table 2).

#### 2.2.3. CSF Glutamate Level

The results of the 3-way ANOVA revealed that the treatment had a significant primary effect on both CSF glutamate (F_3,199_ = 224, *p* < 0.01, _p_η^2^ = 0.789) and BBB disruption (F_3,199_ = 157, *p* < 0.01, _p_η^2^ = 0.723). Using a Bonferroni test for post-hoc analysis, we observed significantly higher levels of CSF glutamate in several groups, including those receiving glutamate doses of 0.423 g/kg (129 µM/L ± 32 µM/L, *p* < 0.01), 0.845 g/kg (162 µM/L ± 43 µM/L, *p* < 0.01), and 1.69 g/kg (142 µM/L ± 93 µM/L, *p* < 0.01), as well as those undergoing BBB opening procedures with TBI at 6 h (107 µM/L ± 87 µM/L, *p* < 0.01), TBI at 24 h (85 µM/L ± 70 µM/L, *p* < 0.01), and HOBBB (142 µM/L ± 75 µM/L, *p* < 0.01), compared to the control group (4 µM/L ± 2.8 µM/L). Sex or interaction between sex and severity of BBB disruption or treatment regarding CSF glutamate levels were not found to have a significant effect (F < 1 in all cases). The data are measurements in µM/L and expressed as mean ± SD (see Table 3). Because there was no effect of sex on any of the dependent variables (Evans blue extravasation index, blood and CSF glutamate level), the male and female groups were combined to obtain greater power in the statistical analysis. For further comparison between the groups, we used a one-factor ANOVA, the results of which are shown below.

### 2.3. Assessment of BBB Disruption 

**Figure 1:**
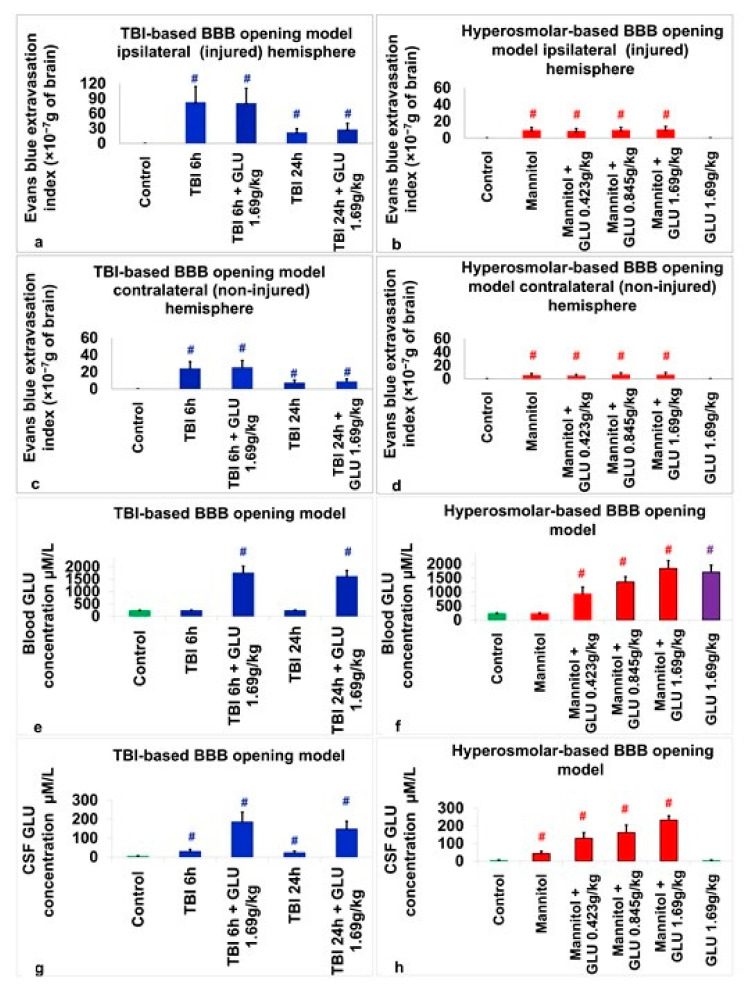
Changes in the brain and blood glutamate concentrations and outcomes in histological assessments of BBB breakdown in study groups compared to control groups. (**a**) Evans blue extravasation index in the injured hemisphere between groups for the TBI-based BBB opening model. (**b**) Evans blue extravasation index in the injured hemisphere between groups for the hyperosmolar-based BBB opening model. (**c**) Evans blue extravasation in the non-injured hemisphere between groups for the TBI-based BBB opening model. (**d**) Evans blue extravasation index in the non-injured hemisphere between groups for the hyperosmolar-based BBB opening model. (**e**) Blood glutamate concentrations between groups for the TBI-based BBB opening model. (**f**) Blood glutamate concentrations between groups for the hyperosmolar-based BBB opening model. (**g**) CSF glutamate concentrations between groups for the TBI-based BBB opening model. (**h**) CSF glutamate concentrations between groups for the hyperosmolar-based BBB opening model. The colors used in the figure correspond to different experimental groups. Specifically, green represents the control group, red corresponds to groups where BBB disruption was induced using the hyperosmolar-based BBB opening model, blue represents groups where BBB disruption was induced through the TBI-based BBB opening model, and purple represents the group that received intravenous glutamate but did not undergo BBB disruption. # *p* < 0.01.

**Figure 2:**
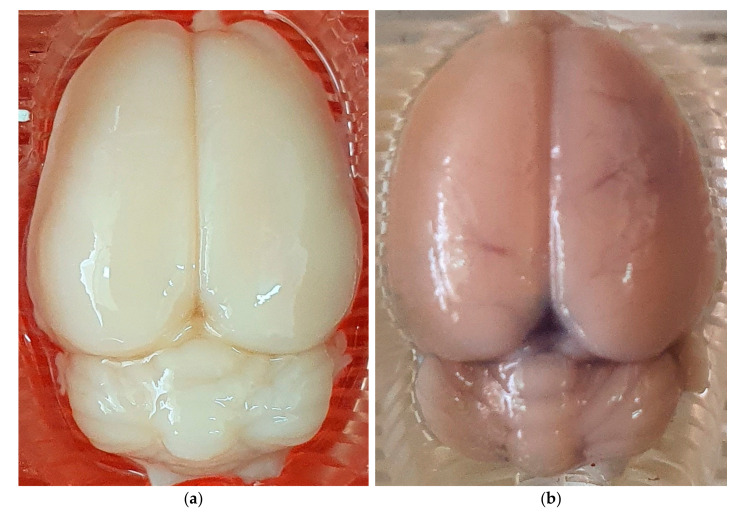

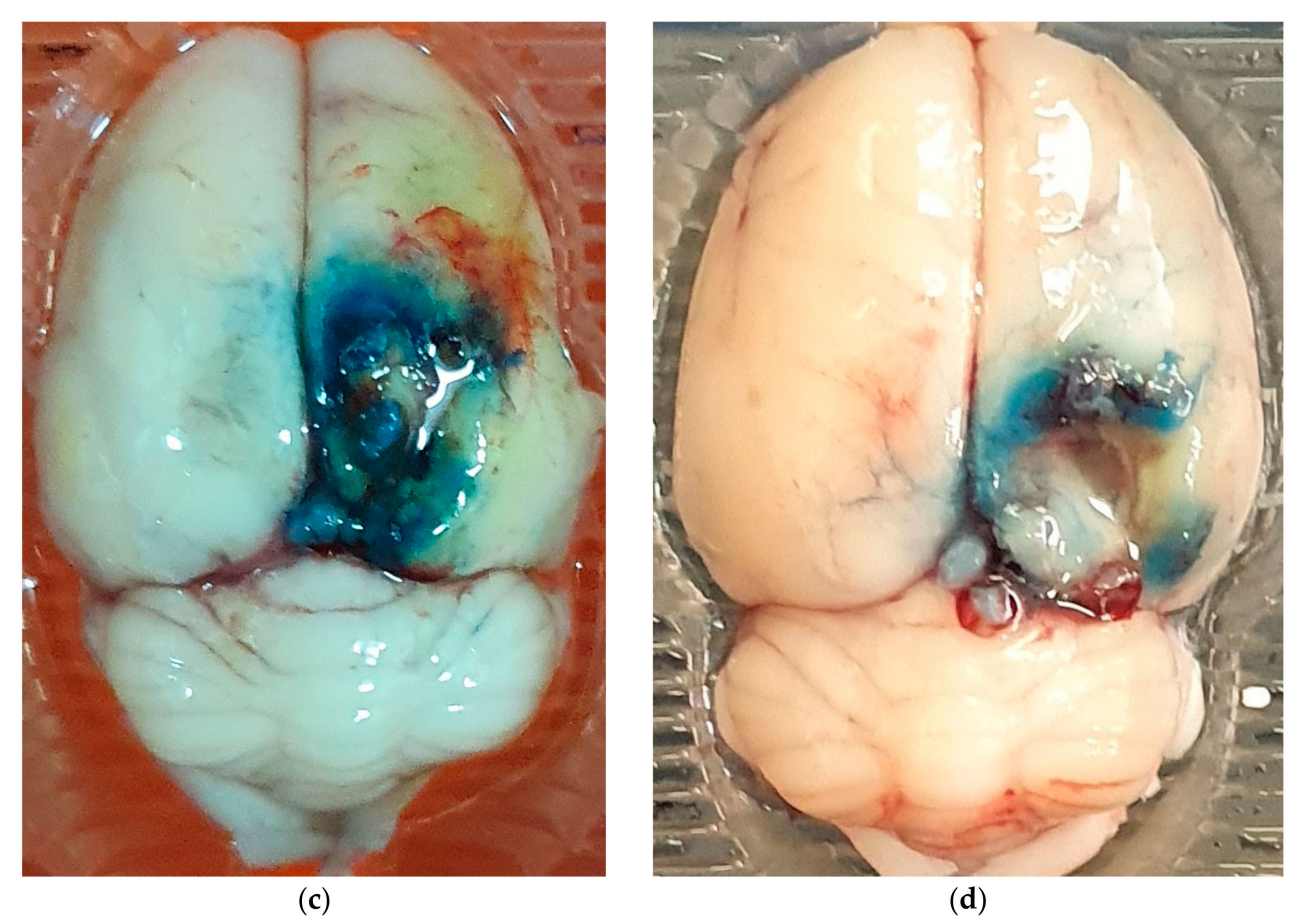
Representative morphological images for hyperosmolar and TBI-based BBB opening models. Injured hemisphere on the right side. (**a**) Representative image of the brain without BBB opening; (**b**) representative brain image for HOBBB model; (**c**) representative brain image for 6 h TBI-based BBB opening model; (**d**) representative brain image for 24 h TBI-based BBB opening model.

Within the injured hemisphere, a one-way ANOVA pointed to a significant difference in the Evans blue extravasation index between the groups belonging to the TBI-based BBB opening model (F_4,95_ = 67.4, *p* < 0.01, η2 = 0.739). Tukey’s post-hoc analysis indicated that the control group (−0.055 × 10^−7^ g ± 0.187 × 10^−7^ g) had significantly lower Evans blue extravasation index than the TBI-based BBB opening groups with TBI at 6 h (83 × 10^−7^ g ± 31 × 10^−7^ g, *p* < 0.01), TBI at 6 h + glutamate 1.69 g/kg (81 × 10^−7^ g ± 29.7 × 10^−7^ g, *p* < 0.01), TBI 24 h (22 × 10^−7^ g ± 7.4 × 10^−7^ g, *p* < 0.01) and TBI at 24 h + glutamate 1.69 g/kg (28 × 10^−7^ g ± 12.1 × 10^−7^ g, *p* < 0.01, see Figure 1a and Figure 2). A significant difference in the Evans blue extravasation index between the groups belonging to the HOBBB model was observed through a one-way ANOVA (F_5,114_ = 68.1, *p* < 0.01, η2 = 0.749). Tukey’s post-hoc analysis revealed that the control group (−0.055 × 10^−7^ g ± 0.187 × 10^−7^ g) had a significantly lower Evans blue extravasation index than the HOBBB groups given mannitol (9.5 × 10^−7^ g ± 3.24 × 10^−7^ g, *p* < 0.01), mannitol + glutamate 0.423 g/kg (8.5 × 10^−7^ g ± 2.9 × 10^−7^ g, *p* < 0.01), mannitol + glutamate 0.845 g/kg (9.7 × 10^−7^ g ± 3.2 × 10^−7^ g, *p* < 0.01) and mannitol + glutamate 1.69 g/kg (10.3 × 10^−7^ g ± 3.7 × 10^−7^ g, *p* < 0.01). The glutamate 1.69 g/kg (0.06 × 10^−7^ g ± 0.13 × 10^−7^ g) group was not different from the control group (see Figure 1b and Figure 2).

For the non-injured hemisphere, a one-way ANOVA indicated a significant difference in the Evans blue extravasation index between the groups belonging to the TBI-based BBB opening model (F_4,95_ = 89.3, *p* < 0.01, η2 = 0.79). According to Tukey’s post-hoc analysis, the control group (0.039 × 10^−7^ g ± 0.175 × 10^−7^ g) had a significantly lower Evans blue extravasation index than the TBI-based BBB opening groups with TBI at 6 h (24.4 × 10^−7^ g ± 7.8 × 10^−7^ g, *p* < 0.01), TBI at 6 h + glutamate 1.69 g/kg (25.7 × 10^−7^ g ± 7.9 × 10^−7^ g, *p* < 0.01), TBI at 24 h (7.6 × 10^−7^ g ± 3.1 × 10^−7^ g, *p* < 0.01) and TBI at 24 h + glutamate 1.69 g/kg (8.9 × 10^−7^ g ± 3 × 10^−7^ g, *p* < 0.01) (see Figure 1c and Figure 2). A one-way ANOVA indicated a significant difference in the Evans blue extravasation index between the groups belonging to the HOBBB model (F_5,114_ = 33.9, *p* < 0.01, η2 = 0.598). According to Tukey’s post-hoc analysis, the control group (0.039 × 10^−7^ g ± 0.175 × 10^−7^ g) had a significantly lower Evans blue extravasation index than the HOBBB groups given mannitol (5.3 × 10^−7^ g ± 2.7 × 10^−7^ g, *p* < 0.01), mannitol + glutamate 0.423 g/kg (4.6 × 10^−7^ g ± 2.2 × 10^−7^ g, *p* < 0.01), mannitol + glutamate 0.845 g/kg (6.3 × 10^−7^ g ± 2.8 × 10^−7^ g, *p* < 0.01), and mannitol + glutamate 1.69 g/kg (6.1 × 10^−7^ g ± 3.3 × 10^−7^ g, *p* < 0.01). The glutamate 1.69 g/kg (0.059 × 10^−7^ g ± 0.1 × 10^−7^ g) group was not different from the control group (see Figure 1d and Figure 2).

### 2.4. Assessment of Blood Glutamate 

A one-way ANOVA indicated a significant difference in the blood glutamate level between the TBI-based BBB opening study groups (F_4,95_ = 451, *p* < 0.01, η2 = 0.95). Tukey’s post-hoc analysis showed a significantly higher level of blood glutamate for the treatment of TBI at 6 h + glutamate 1.69 g/kg (1749 µM/L ± 280 µM/L, *p* < 0.01) and TBI at 24 h + glutamate 1.69 g/kg (1609 µM/L ± 245 µM/L, *p* < 0.01) compared to the control rats (224 µM/L ± 26 µM/L). The data are measurements in µM/L and expressed as mean ± SD (see Figure 1e). A one-way ANOVA indicated a significant difference in the blood glutamate level between the HOBBB study groups (F_5,114_ = 245, *p* < 0.01, η2 = 0.915). Tukey’s post-hoc analysis showed a significant higher level of blood glutamate for the treatment of mannitol + glutamate 0.423 g/kg (927 µM/L ± 251 µM/L, *p* < 0.01), mannitol + glutamate 0.845 g/kg (1361 µM/L ± 189 µM/L, *p* < 0.01), mannitol + glutamate 1.69 g/kg (1837 µM/L ± 287 µM/L, *p* < 0.01) and glutamate 1.69 g/kg (1707 µM/L ± 254 µM/L, *p* < 0.01) compared to the control rats (224 µM/L ± 26 µM/L). The data are measurements in µM/L and expressed as mean ± SD (see Figure 1f).

### 2.5. Assessment of CSF Glutamate 

In the TBI-based BBB opening model, a one-way ANOVA revealed a significant difference in the CSF glutamate levels between the experimental groups, and a subsequent Student’s t-test indicated a significant higher level of CSF glutamate in the TBI at 6 h + glutamate 1.69 g/kg (185 µM/L ± 51.7 µM/L, t(38) = 15.7, *p* < 0.01), TBI at 6 h (29 µM/L ± 11.7 µM/L, t(38) = 9.29, *p* < 0.01), TBI at 24 h (22.5 µM/L ± 9.3 µM/L, t(38) = 8.47, *p* < 0.01) and TBI at 24 h + glutamate 1.69 g/kg (147.8 µM/L ± 41.5 µM/L, t(38) = 15.4, *p* < 0.01) groups compared to the control rats (4.1 µM/L ± 2.8 µM/L). The data are measurements in µM/L and expressed as mean ± SD (see Figure 1g). 

In the HOBBB model, a one-way ANOVA revealed a significant difference in the CSF glutamate levels between the experimental groups (F_5,114_ = 290, *p* < 0.01, η2 = 0.927) and a subsequent Tukey post-hoc analysis showed a significant higher level of CSF glutamate for mannitol (43 µM/L ± 13 µM/L, *p* < 0.01), mannitol + glutamate 0.423 g/kg (129 µM/L ± 32 µM/L, *p* < 0.01), mannitol + glutamate 0.845 g/kg (162 µM/L ± 43 µM/L, *p* < 0.01) and mannitol + glutamate 1.69 g/kg (233 µM/L ± 24 µM/L, *p* < 0.01) compared to the control rats (4 µM/L ± 2.8 µM/L). The glutamate 1.69 g/kg (3.7 µM/L ± 2.8 µM/L) group was not different from the control group. The data are measurements in µM/L and expressed as mean ± SD (see Figure 1h).

### 2.6. Correlations between CSF Glutamate, Blood Glutamate, and the Degree of BBB Permeability

**Figure 3:**
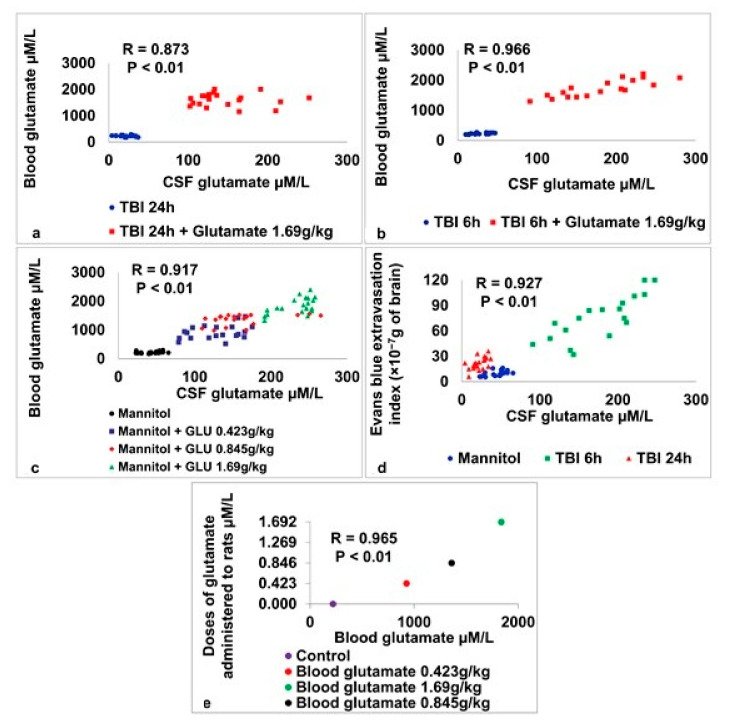
Changes in brain and blood glutamate concentrations and outcomes of histological assessments of BBB breakdown in study groups compared to control groups.

Correlations were determined between CSF glutamate, blood glutamate, and the degree of BBB permeability (see Figure 3a–e). A strong correlation was found between (i) the glutamate concentration in the blood and CSF in rats subjected to a model of BBB destruction as a result of TBI after 24 h (r_p(40)_ = 0.873, *p* < 0.01, see Figure 3a); (ii) the glutamate concentration in the blood and CSF in rats subjected to a model of BBB destruction as a result of TBI after 6 h (r_p(40)_ = 0.966, *p* < 0.01, see Figure 3b); (iii) the glutamate concentration in the blood and CSF in rats subjected to a model of BBB destruction as a result of HOBBB (r_p(80)_ = 0.917, *p* < 0.01, see Figure 3c); (iv) the CSF glutamate concentration and Evans blue extravasation index in rats subjected to a model of BBB destruction as a result of TBI and HOBBB (r_p(60)_ = 0.927, *p* < 0.01, see Figure 3d); (v) rats treated with various doses of glutamate and blood glutamate concentrations (r_s(4)_ = 0.965, *p* < 0.01, see Figure 3e).

## 3. Discussion

In this study, we examined the role of BBB disruption as a key factor that contributes to the excess of glutamate in the CSF. We specifically studied the effect of different concentrations of blood glutamate on several models of BBB disruption, including both (1) an isolated model of BBB disruption known as HOBBB, and (2) traditional TBI (at 6 h and 24 h), which has multiple pathological processes, and compared the study rats to the control rats. The primary conclusion of this study is that while a healthy BBB effectively protects the brain from elevated levels of blood glutamate, a compromised BBB contributes to a pathological excess of glutamate in the CSF, which can account for long-lasting effects of the TBI. 

As expected, the CSF glutamate levels in the group of rats treated with glutamate at a concentration of 1.69/kg, but that were not subjected to induction of BBB destruction, were not significantly different from the levels of CSF glutamate in the control group that did not receive glutamate. This suggests that a healthy BBB effectively protects the brain from high levels of glutamate in the blood. However, the levels of cerebrospinal fluid glutamate in all models of BBB destruction, both with mannitol and with two models of TBI (at 6 h and 24 h), were increased in all experimental groups compared with the control rats. In addition, a strong correlation was documented between (1) the levels of CSF glutamate and blood glutamate and (2) the level of CSF glutamate and the degree of BBB destruction.

After additionally studying the groups with different concentrations of blood glutamate (mannitol, mannitol + glutamate 0.423 g/kg, mannitol + glutamate 0.845 g/kg, mannitol + glutamate 1.69 g/kg), which had the same degree of BBB destruction, we concluded that it is the degree of BBB permeability that regulates the levels of brain glutamate in healthy as well as in injured brains. It was determined that the level of glutamate in the CSF in the mannitol groups with TBI at 6 h, and TBI at 24 h did not exceed 43 µM/L. This finding confirms our hypothesis [15] that a level of 50–60 µM/L seems to be the maximum allowable level of CSF glutamate in a brain injury, since the level of blood plasma glutamate acts as its natural limiter. 

To explore the role of BBB disruption as a key factor that contributes to excess CSF glutamate, in this study we used the following two different rat models, in addition to control groups, to study BBB permeability: (1) a model of HOBBB disruption, and (2) TBI-induced BBB disruption. 

The TBI model was studied at two time points that differed in the severity of BBB destruction. This effect is known as biphasic BBB destruction, the dynamics of which have been studied and described previously [33]. The value of these two TBI models was that they made it possible to study the effects of varying severity in the destruction of the BBB. In the HOBBB disruption model, we induced the same degree of BBB destruction in all groups and tested the effect of different concentrations of blood glutamate on the concentration of brain glutamate.

As expected in the TBI model, the more severe BBB permeability was recorded at 6 h compared to 24 h. Both TBI models, at 6 h and 24 h, showed a higher degree of BBB destruction compared with the HOBBB model. BBB permeability was measured separately in two hemispheres in all the experimental groups. We recorded a significant increase in BBB permeability in the injured hemisphere in the TBI model, and in the hemisphere on the side of the mannitol injection in the HOBBB disruption model compared to the contralateral hemisphere. This has been previously observed in the literature [34,35]. We attribute this significant increase to the influence of factors such as cerebral edema and massive neuronal death, which exacerbate BBB breakdown [34]. Multivariate statistical analysis confirmed that the level of BBB destruction in the experimental groups depended only on the type of model of BBB destruction; no influence of any other factors or interactions between factors were registered on BBB destruction. 

As expected, we did not observe any differences in the blood glutamate concentrations between the groups of rats administered mannitol + glutamate 1.69 g/kg, TBI at 6 h + glutamate 1.69 g/kg, or TBI at 24 h + glutamate 1.69 g/kg, compared to the group with glutamate 1.69 g/kg. There were also no differences in the concentration of glutamate in the blood of rats in the TBI and mannitol groups that did not receive glutamate (mannitol, TBI at 6 h, and TBI at 24 h), compared with the control group. Multivariate statistical analysis confirmed no influence between BBB degradation and blood glutamate levels (see Results). All groups in this study showed a very strong correlation between the concentration of glutamate in the blood and the amount of intravenously administered glutamate r = 0.965.

We conclude that a healthy BBB effectively protects the brain from high levels of blood glutamate. In addition, after BBB disruption, the concentration of CSF glutamate is strongly correlated with the concentration of blood glutamate and relies on the degree of disruption of the BBB; the permeability of the BBB is a central component in regulating the levels of glutamate in the brain. 

## 4. Materials and Methods

### 4.1. Animals

The experiments were sanctioned by the Animal Care Committee of Ben-Gurion University of the Negev (Beer-Sheva, Israel). This experiment used 100 male and 100 female (for a total of 200) Sprague-Dawley rats (Harlan Laboratories, Jerusalem, Israel), all weighing between 300 and 350 g. Purina chow (Ssniff Low-Phytoestrogen breeding diet, Soest, Germany) and water were made available ad libitum. Rats were situated in environmentally controlled rooms at a temperature of 22 °C, with a 12 h light/12 h dark cycle. All the tests took place between 8 am and 6 pm.

### 4.2. Drugs

Evans Blue (E2129) and glutamate (G1251) were purchased from Merck & Co. Inc. (Rahway, NJ, USA). In addition, 25% mannitol was purchased from Hospira, Inc. (Lake Forest, IL, USA).

### 4.3. Experimental Design

All rats were randomly separated into 10 groups of 20 rats each (10 males and 10 females) to undergo hyperosmolar opening of the BBB (HOBBB), TBI, or sham operation, with a variety of treatment methods (see below for HOBBB and TBI protocols). The effects of BBB breakdown on high concentrations of cerebral glutamate were studied in rats using the following 10 treatment groups: (1) HOBBB (induced by administration of mannitol 25% 2.4 cc into intra-carotid artery); (2) HOBBB + intravenous (IV) glutamate 1.69 g/kg (85); (3) HOBBB + IV glutamate 0.845 g/kg; (4) HOBBB + IV glutamate 0.423 g/kg; (5) TBI-6 h; (6) TBI after 6 h + IV glutamate 1.69 g/kg; (7) TBI at 24 h; (8) TBI at 24 h + IV glutamate 1.69 g/kg; (9) IV glutamate 1.69 g/kg; (10) sham-operated rats. 

Immediately after HOBBB induction and 5 h and 23 h after TBI, the rats were intravenously administered 2% Evans blue at 4 mL/kg. One hour later, blood and CSF were collected to determine the concentration of glutamate. Rats were euthanized, and the brains were collected to assess BBB breakdown. NSS was assessed in the TBI and control groups 5 h after the injury.

### 4.4. HOBBB

**Figure 4:**
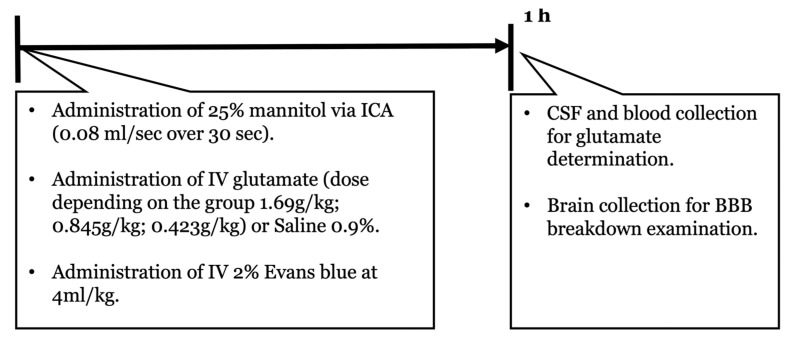
A timeline protocol for hyperosmolar opening of the BBB. Intravenous (IV); internal carotid artery (ICA).

The rats were anesthetized with 5% inhaled isoflurane and maintained at 1.5–2.5%, with 50% medical air and 50% oxygen, a tail vein catheter was placed, and a catheter was inserted into the internal carotid artery (ICA) [36]. Mannitol 25% was perfused through the ICA at the rate of 0.08 mL/s over 30 s [37]. Afterwards, glutamate, or a similar volume of normal saline solution, was administered through the tail with an intravenous catheter. A timeline protocol for the HOBBB-based BBB opening model can be observed in Figure 4. 

### 4.5. TBI

**Figure 5:**
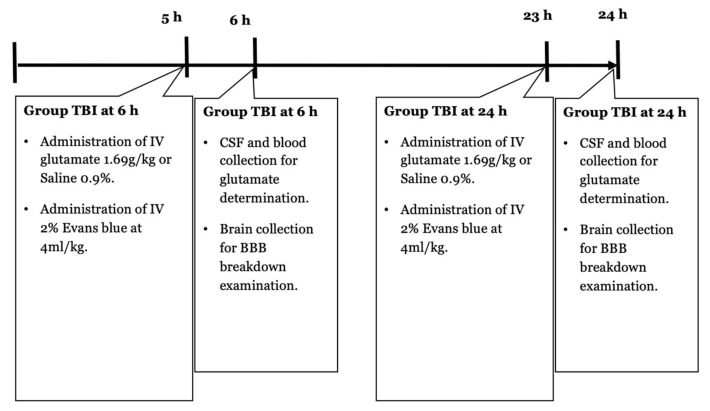
A timeline protocol for TBI-based BBB opening model. Intravenous (IV).

TBI was performed as previously described [34,38]. Rats were anesthetized with 5% inhaled isoflurane and maintained at 1.5–2.5%, with 50% medical air and 50% oxygen. Before the incision, the scalp was injected with 0.5% bupivacaine. The scalp was then incised and reflected laterally with the left temporal muscle. The underlying periosteum was then also dissected to expose the skull. A craniotomy (4 mm lateral and 4 mm posterior to bregma) was performed at 5 mm with a trephine (Roboz Surgical Instrument Co., Gaithersburg, MD, USA) attached to the drill bit of an electrical drill (Stoelting, Wood Dale, IL, USA). A Luer 3-way stopcock was secured in place by cyanoacrylate adhesive and dental acrylic. The injury was then caused by a pressure pulse of 2.2 atmospheres. TBI was induced by a fluid-percussion device over 21–23 ms through the 3-way stopcock. The fluid pulse from the piston plunger through the pendulum was enabled by saline flowing continuously into the dura to ensure efficient transmission of the pressure pulse. Rats in the sham-operated group underwent the same procedure but did not receive a craniotomy. After the TBI was induced, the incision was closed, and the rats recovered from the anesthesia. This experiment involved 2 control groups of rats that did not have BBB disruption, including one group that received intravenous saline solution and another group that received intravenous glutamate at a dose of 1.69 g/kg. A timeline protocol for the TBI-based BBB opening model can be observed in Figure 5.

### 4.6. Blood Sample Collection

Blood glutamate levels were analyzed by collecting blood from the tail vein using a 24-gauge Neoflon (Becton Dickinson, Helsingborg, Sweden) catheter, which was subsequently removed from the vein [39].

### 4.7. Determination of Blood Glutamate

Whole blood (200 μL aliquot) was deproteinized by mixing an equal volume of ice cold 1 M perchloric acid and then centrifuging at 10,000× *g* for 10 min at 4 °C. The pellet was discarded and the supernatant was collected, adjusted to pH 7.2 with 2 M K_2_CO_3_, and stored at −80 °C for later analysis. The concentration of glutamate was measured utilizing the fluorometric method [40]. A 60 μL aliquot from the perchloric acid supernatant was combined with 90 μL of a 0.3 M glycine and 0.25 M hydrazine hydrate buffer and adjusted to a pH of 8.6 with 1 M H_2_SO_4_ and contained 11.25 U of glutamate dehydrogenase in 10 mM nicotinamide adenine dinucleotide. After an incubation period of 30 to 45 min at room temperature, the fluorescence was measured at 460 nm with excitation at 350 nm. A standard curve was ascertained with glutamate concentrations ranging from 0 to 6 mM. Measurements were performed at least in duplicates [40].

### 4.8. Cerebrospinal Fluid Collection

Rats were anesthetized with 5% inhaled isoflurane and maintained at 1.5–2.5%, with 50% medical air and 50% oxygen, the cisterna magna was cannulated, as previously described [41], and 0.1 to 0.2 mL of CSF was gently aspirated.

### 4.9. Cerebrospinal Fluid Glutamate

Perchloric acid (25 μL) of 0.3 M was added to freshly obtained CSF (110 μL), and then centrifuged at 10,000× *g* for 10 min at 4 °C. The supernatant was obtained, adjusted to pH 7.2 with 12.5 μL of 2 M K_2_CO_3_ and stored at −80 °C for later analysis [42,43]. Analysis was performed by a fluorometric method, as described above for the blood samples. 

### 4.10. Assessment of BBB Disruption

The samples of brain slices were weighed and homogenized in trichloroacetic acid, using the formula of 1 g of brain tissue in 4 mL of 50% trichloroacetic acid. It was then centrifuged at 10,000× *g* for 20 min and the supernatant was diluted 1:3 with 96% ethanol. A fluorescence detector was employed at a 620 nm excitation wavelength (bandwidth 10 nm) and 680 nm emission wavelength (bandwidth 10 nm) [34,44]. 

### 4.11. Neurological Performance

Rats were assessed for their neurological status as previously described [34,45] using the Neurological Severity Score (NSS). Two blinded observers assigned points for several criteria that were combined for an overall score of 0–25, with a score of 25 for the highest neurological deficiency and 0 for intact neurological status. Specifically, the assessed criteria included the following: the ability to leave a circle (3 point scale), gait on a wide surface (3 point scale), gait on a narrow surface (4 point scale), ability to stay on a narrow surface (2 point scale), reflexes (5 point scale), seeking behavior (2 point scale), beam walking (3 point scale), and beam balance (3 point scale) [34,45].

### 4.12. Statistical Analysis

Statistical evaluation was carried out using the SPSS-22 package (SPSS Inc., Chicago, IL, USA). A Kolmogorov–Smirnov test determined the correct test for comparisons between the different parameters. The data were analyzed with 3-way ANOVA for the effects of sex, BBB disruption, and treatment. Post-hoc Bonferroni tests or planned pair comparisons were performed when the interactions were significant. We ascertained the significance of the comparisons between the groups with the one-way ANOVA with a subsequent Tukey’s post-hoc test or Student’s t-tests. Using Pearson’s test (for parametric data) and Spearman’s test (for non-parametric data), we calculated the correlation between CSF and blood glutamate levels and BBB disruption. The NSS were compared by the Mann–Whitney U test. Normally distributed data and continuous variables were presented as mean ± SD. Non-parametric data were presented as median ± interquartile range. The results were considered statistically significant when *p* < 0.05.

## 5. Conclusions

Through our multi-modal approach to inducing and analyzing BBB disruption, this study explored the effects of BBB breakdown on glutamate levels in the brain and blood. We found that a healthy BBB is a critical factor in protecting the brain from excess levels of blood glutamate. Additionally, we observed that after BBB disruption, CSF glutamate concentration is linked to blood glutamate concentration, which are dependent on the severity of BBB disruption. The permeability of the BBB provides a pathway to modulating glutamate levels in the brain.

We hope that these observations will contribute to new approaches in the treatment of the consequences of TBI and other diseases where long-term disruption of the BBB is the central mechanism of their development. Chronic BBB disruption is a novel idea, and we propose that it has significant impact on the consequences of brain injury. 

## Figures and Tables

**Table 1:** Main effects of the model of BBB disruption, treatment and sex and their interaction effects on the Evans blue extravasation index. No significance (n-s).

Effect	TBI 6 h vs. Control	TBI 24 h vs. Control	HOBBB vs. Control
Models of BBB disruption	*p* < 0.01	*p* < 0.01	*p* < 0.01
Treatment	n-s	n-s	n-s
Sex	n-s	n-s	n-s
Models of BBB disruption × treatment	n-s	n-s	n-s
Models of BBB disruption × sex	n-s	n-s	n-s
Models of BBB disruption × sex × treatment	n-s	n-s	n-s
Treatment × sex	n-s	n-s	n-s

**Table 2:** Main effects of model of BBB disruption, treatment and sex and their interaction effects on blood glutamate level. No significance (n-s).

Effect	Glutamate 0.423 g/kg vs. Placebo	Glutamate 0.845 g/kg vs. Placebo	Glutamate 1.69 g/kg vs. Placebo
Models of BBB disruption	n-s	n-s	n-s
Treatment	*p* < 0.01	*p* < 0.01	*p* < 0.01
Sex	n-s	n-s	n-s
Models of BBB disruption × treatment	n-s	n-s	n-s
Models of BBB disruption × sex	n-s	n-s	n-s
Models of BBB disruption × sex × treatment	n-s	n-s	n-s
Treatment × sex	n-s	n-s	n-s

**Table 3:** Main effects of the model of BBB disruption, treatment and sex and their interaction effects on CSF glutamate level. No significance (n-s).

Effect	Glutamate 0.423 g/kg vs. Placebo	Glutamate 0.845 g/kg vs. Placebo	Glutamate 1.69 g/kg vs. Placebo	TBI 6 h vs. Control	TBI 24 h vs. Control	HOBBB vs. Control
Models of BBB disruption	*p* < 0.01	*p* < 0.01	*p* < 0.01	*p* < 0.01	*p* < 0.01	*p* < 0.01
Treatment	*p* < 0.01	*p* < 0.01	*p* < 0.01	*p* < 0.01	*p* < 0.01	*p* < 0.01
Sex	n-s	n-s	n-s	n-s	n-s	n-s
Models of BBB disruption × treatment	*p* < 0.01	*p* < 0.01	*p* < 0.01	*p* < 0.01	*p* < 0.01	*p* < 0.01
Models of BBB disruption × sex	n-s	n-s	n-s	n-s	n-s	n-s
Models of BBB disruption × sex × treatment	n-s	n-s	n-s	n-s	n-s	n-s
Treatment × sex	n-s	n-s	n-s	n-s	n-s	n-s

## Data Availability

Data are available upon reasonable request.
